# Supplementary material for: Stereotactic Ablative Radiotherapy for Oligometastatic Hepatocellular Carcinoma: A Multi-Institutional Retrospective Study (KROG 20-04)
Source: Cancers (Basel). 2022 Nov 27;14(23):5848. doi: 10.3390/cancers14235848 (PMC9735640; doi:10.3390/cancers14235848)
Supplement: Supplementary file 1 [file cancers-14-05848-s001.zip › cancers-2044777-supplementary.pdf]

Supplementary Table S1. Oligometastatic bone lesions

Total bone lesions	n	%
C spine	5	9%
T spine	15	27%
L spine	18	33%
Pelvic bone	10	18%
Rib	6	11%
Other	1	2%
Fraction dose (Gy)		
6-9	19	35%
10-14	7	13%
15	29	53%
Total dose (Gy)		
≤40	21	50%
40-50	6	14%
50-60	28	67%

Supplementary Table S2. Oligometastatic lung lesions

Total lung lesions	n	%
RUL	6	14%
RML	4	10%
RLL	12	29%
LUL	8	19%
LLL	12	29%
Fraction dose (Gy)		
6-9	4	10%
10-14	14	33%
15	24	57%
Total dose (Gy)		
≤40	3	7%
40-50	6	14%
50-60	33	79%
